# Presence of bacterial infection and duration of antibiotic therapy in patients with standardized sepsis detection in the emergency department

**DOI:** 10.1186/cc14032

**Published:** 2014-12-03

**Authors:** TC Minderhoud, C Spruyt, M Lomax, S Huisman, SCE Schuit, M-D Levin

**Affiliations:** 1 Department of Internal Medicine, Albert Schweitzer Ziekenhuis, Dordrecht, the Netherlands; 2 Department of Internal Medicine and Emergency Medicine, Erasmus University Medical Center, Rotterdam, the Netherlands

## Introduction

In 2002 the international Surviving Sepsis Campaign was initiated. Following this, Dutch authorities introduced a nationwide safety campaign, encouraging screening for sepsis and advocating early treatment. In 2010, the Albert Schweitzer hospital, a large community hospital, introduced a screening program for sepsis in the emergency department (ED) [1]. The goal of this study was to evaluate the bacterial outcome in patients targeted with this campaign.

## Methods

All patients 18 years and older visiting the ED were screened using the criteria of systemic inflammatory response syndrome (SIRS). Patients with more than two SIRS criteria and a clinical suspicion for infection were eligible for prompt antibiotic administration, after a short assessment by an ED physician. Patient data were collected prospectively, but a retrospective analysis was conducted using a cohort of patients presenting in the ED in the first 6 months of 2011. The definitions for sepsis severity were derived from the guidelines of the Surviving Sepsis Campaign in 2008 and criteria to define bacterial infection were derived from an article by Limper and colleagues [2].

## Results

A total of 269 patients were included in the study. Review of infectious outcomes showed no evidence of bacterial disease in 79 (29.4%) patients. Of these patients, 70.9% were in the lowest category of sepsis (SIRS and clinical suspicion of infectious disease). Patients in the lowest category were less likely to suffer from bacterial infection than patients (*P *= 0.046, see Table 1). In the patients without objective bacterial infection, the median duration of antibiotic treatment was 7 days (IQR 4 to 10). Overall mortality was 7.8 %, which is low compared to current literature regarding (severe) sepsis [3,4], but comparable to literature addressing SIRS and fever in the ED settings [5-8].

**Table 1:** 

	No bacterial infection (*n *= 79)	Bacterial infection proven/probable (*n *= 190)	*P *value
Culture-proven infection	-	97 (51.1%)	-
Positive blood cultures	-	51 (26.8%)	-
Probable bacterial infection	-	93 (48.9 %)	-
Sepsis category			
Sepsis	56 (70,9%)	110 (57.9%)	0.046^a,b^
Severe sepsis	20 (26,3%)	57 (30.0%)	0.549^a^
Sepsis-induced hypotension	2 (2,6%)	18 (9.5%)	0.053^a,b^
Septic shock	1 (1,3%)	5 (2.6%)	0.506^a^
Antibiotic treatment duration (days)^c^	7 (4 to 10)	10 (7 to 14)	0.000^b,d^

^a^Chi-square test. ^b^Test reached statistical significance. ^c^Data provided as median (IQR). ^d^Mann-Whitney *U *test.

## Conclusion

Much effort has been put into promoting early antibiotic treatment for (bacterial) sepsis. However, overtreatment has hardly been addressed and no optimal screening strategy has been identified. Evaluation of our screening protocol using SIRS criteria showed that almost 30% of patients did not suffer from bacterial infection but did receive antibiotic treatment for a median duration of 7 days. Future investigations should address the possible negative effects of overtreatment.
